# Mosquito-associated Dengue Virus, Key West, Florida, USA, 2010

**DOI:** 10.3201/eid1711.110419

**Published:** 2011-11

**Authors:** Amanda S. Graham, Catherine A. Pruszynski, Lawrence J. Hribar, David J. DeMay, Adriane N. Tambasco, Anne E. Hartley, Edsel M. Fussell, Scott F. Michael, Sharon Isern

**Affiliations:** Florida Gulf Coast University, Fort Myers, Florida, USA (A.S. Graham, A.E. Hartley, S.F. Michael, S. Isern); Florida Keys Mosquito Control District, Key West, Florida, USA (C.A. Pruszynski, L.J. Hribar, D.J. DeMay, A.N. Tambasco, E.M. Fussell)

**Keywords:** dengue, phylogeny, vector-borne infections, zoonoses, Key West, Florida, mosquito, RT-PCR, envelope, viruses, letter

**To the Editor:** Except for sporadic cases along the Texas–Mexico border, local transmission of dengue virus (DENV) has not occurred in the contiguous United States since 1946. In 2009, DENV was diagnosed in a vacationer to Key West, Florida (*1*). Subsequently, 25 other cases were reported that year, transmission was confirmed by detection of DENV serotype 1 (DENV-1) in local mosquitoes, and a random serosurvey showed evidence of recent DENV infection in 5.4% of Key West residents (*1*). Transmission continued in 2010, and an additional 63 cases were confirmed (*2*). We used PCR amplification and sequence analysis of virus identified from mosquito collections during 2010 to identify the closest relatives, probable geographic origin, and divergence time of the Key West DENV.

A total of 1,178 pools of *Aedes aegypti* mosquitoes were collected in Monroe County, Florida, during January 27–December 17, 2010 (Figure A1, panel A), by using BG-Sentinel (Biogents, Regensburg, Germany) or CDC (Clarke, Roselle, IL, USA) light traps, and stored at −80°C. Reverse transcription PCR was conducted on each pool by using primers designed to amplify all 4 DENV serotypes, followed by seminested PCR with serotype-specific primers (*3*). Results from 2 Key West mosquito pools collected on June 25 and 30 showed a positive first-round reverse transcription PCR and a positive second-round PCR specific for DENV-1. A third Key West pool collected on August 27 showed only a positive second-round PCR specific for DENV-1. No other DENV serotypes were detected.

DENV-1–specific primers (5′-GGGCCTTGAGACACCCAGG-3′ and 5′-CCTCCCATGCCTTCCCAATGGC-3′) were used to amplify products encompassing the envelope (E) gene region and parts of the premembrane and nonstructural 1 genes from the pools collected on June 25 and 30. PCR products were sequenced by using amplification and internal primers to provide double or triple coverage (Functional Biosciences, Madison, WI, USA). The sequences from these 2 pools were identical (GenBank accession no. JF519855). We used ClustalX (*4*) to align the Key West sequences with 175 nonredundant American DENV-1 sequences from the National Center for Biotechnology Information Virus Variation database (*5*) and 9 additional DENV-1 subgenomic E sequences from GenBank, which provided a comprehensive set of American DENV-1 sequences, including several isolates from Hawaii (USA) and Easter Island (Chile) that grouped with Asian DENV-1 clades as an outgroup. Maximum-likelihood phylogenetic analysis of the 1,484-nt E gene region was conducted by using SeaView software (*6*).

The analysis showed that American DENV-1 strains clustered by geography and by year of collection (Figure A1, panel B). This clustering might be the result of lineage replacement that has been described in DENV-1 (*7*). Additionally, clustering might be influenced by serotype prevalence or sampling bias. For example, the sequence database contains few Caribbean isolates from after 2000 and few Central or South American isolates from before 2000. The Key West sequence grouped as a member of a large clade of recent viruses from Central America that was separated from Caribbean and South American viruses with a well-supported bootstrap value (86%) and relatively long branch length. The closest relatives were 2 strains isolated in Nicaragua in 2006 and 2008. The bootstrap support for this grouping was 100%. Phylogenetic analyses with neighbor-joining, maximum-parsimony, and Bayesian methods gave trees with similar topologies, including clear separation of most recent Central American isolates into 1 clade, as well as grouping of the Key West sequence with the same 2 isolates from Nicaragua (data not shown). No protein coding changes between these strains were identified, which suggests purifying selection for an optimum phenotype. There were 8 synonymous differences over the 1,708-nt amplified region between the Key West and Nicaragua 2008 sequences. Previous molecular clock determinations for DENV-1 provided a range of 2.5–7.0 × 10^–4^ substitutions per nucleotide per year (*8*). This calculation produced an estimate of a 6.7–18.7-year divergence time between the Key West virus and the most closely related Nicaragua strain. When during this time the ancestor of Key West DENV was introduced to Florida is unknown.

Analysis of the entire Key West DENV-1 genome may help pinpoint the origin and address the possibility of selective pressure on other genes or recombination events (*9*). Given the recent reports of DENV in residents of other Florida counties who had no travel histories (*2*), monitoring of Key West and other nearby urban areas for evidence of local DENV transmission should continue.

## Figures and Tables

**Figure A1:**
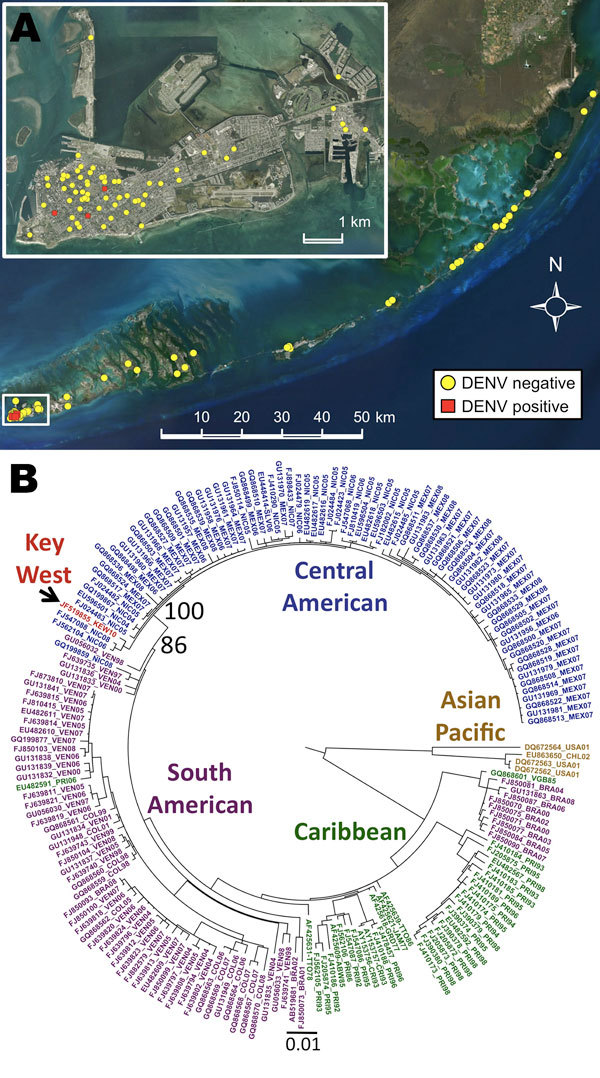
A) Mosquito collection locations, Key West, Florida, USA, January 27–December 17, 2010. Inset shows Key West. Red squares indicate locations of dengue virus serotype 1 (DENV-1)–positive mosquito pools. Yellow circles indicate locations of negative mosquito pools. The longitude and latitude of Key West are 24°33′20.53′′ N, 81°46′57.33′′W. B) Maximum-likelihood phylogenetic tree of the 1,484-nt envelope gene region from American DENV-1 isolates. Sequences are labeled with GenBank accession numbers, country, and year. Countries are given as standard 3-letter codes. Scale bar indicates number of substitutions per site. The Key West sequence is shown in red; Central American sequences, in blue; Caribbean sequences, in green; South American sequences, in purple, Asian/Pacific sequences, in gold. Percentage bootstrap values are shown for the nodes that separate the Central American clade (86%) and for the node that separates the clade containing the Key West virus and its 2 closest relatives (100%). Isolates from Hawaii (USA) and Easter Island (CHI) are of Asian origin and form an outgroup. Viruses group by geography and by year. Selected percentage bootstrap values are shown. Tree was drawn by using FigTree software (http://tree.bio.ed.ac.uk/software/figtree).

## References

[1] Centers for Disease Control and Prevention. Locally acquired dengue—Key West, Florida, 2009–2010. MMWR Morb Mortal Wkly Rep. 2010;59:577–81. 20489680

[2] Anil L, Stanek D, Blackmore C, Stark L, Mock V. Florida arbovirus surveillance week 52: December 26–January 1, 2011 [cited 2011 Mar 18]. http://www.doh.state.fl.us/Environment/medicine/arboviral/pdfs/2010/2010Week52ArbovirusReport_1_1_2011.pdf

[3] Lanciotti RS, Calisher CH, Gubler DJ, Chang GJ, Vorndam AV. Rapid detection and typing of dengue viruses from clinical samples by using reverse transcriptase–polymerase chain reaction. J Clin Microbiol. 1992;30:545–51. 1372617 10.1128/jcm.30.3.545-551.1992 PMC265106

[4] Thompson JD, Gibson TJ, Plewniak F, Jeanmougin F, Higgins DG. The ClustalX windows interface: flexible strategies for multiple sequence alignment aided by quality analysis tools. Nucleic Acids Res. 1997;24:4876–82. 10.1093/nar/25.24.4876 PMC147148 9396791

[5] Resch W, Zaslavsky L, Kiryutin B, Rozanov M, Bao Y, Tatusova TA. Virus variation resources at the National Center for Biotechnology Information: dengue virus. BMC Microbiol. 2009;9:65. 10.1186/1471-2180-9-65 19341451 PMC2675532

[6] Gouy M, Guindon S, Gascuel O. SeaView version 4: a multiplatform graphical user interface for sequence alignment and phylogenetic tree building. Mol Biol Evol. 2010;27:221–4. 10.1093/molbev/msp259 19854763

[7] Zhang C, Mammen MP Jr, Chinnawirotpisan P, Klungthong C, Rodpradit P, Monkongdee P, Clade replacements in dengue virus serotypes 1 and 3 are associated with changing serotype prevalence. J Virol. 2005;79:15123–30. 10.1128/JVI.79.24.15123-15130.2005 16306584 PMC1316048

[8] Twiddy SS, Holmes EC, Rambaut A. Inferring the rate and time-scale of dengue virus evolution. Mol Biol Evol. 2003;20:122–9. 10.1093/molbev/msg010 12519914

[9] Carvalho SES, Martin DP, Oliveira LM, Ribeiro BM, Nagata T. Comparative analysis of American dengue virus type 1 full-genome sequences. Virus Genes. 2010;40:60–6. 10.1007/s11262-009-0428-0 19997970

